# Fluorescence Nanoscopy of Single DNA Molecules by Using Stimulated Emission Depletion (STED)**

**DOI:** 10.1002/anie.201100371

**Published:** 2011-05-06

**Authors:** F Persson, P Bingen, T Staudt, J Engelhardt, J O Tegenfeldt, Stefan W Hell

**Affiliations:** Department of Physics, University of GothenburgFysikgränd 3, 412 96 Gothenburg (Sweden); Optical Nanoscopy Division, German Cancer Research Center (DKFZ)Im Neuenheimer Feld 280, 69120 Heidelberg (Germany), Fax: (+49) 6221-54-51210

**Keywords:** DNA, dyes/pigments, fluorescence, single-molecule studies, stimulated emission depletion (STED)

Lens-based (far-field) fluorescence microscopy has played a key role in the life sciences, but for most of the time the resolution has been limited to about Δ*r*=*λ*/(*2*ΝΑ)>200 nm, with λ denoting the wavelength of light and NA the numerical aperture of the lens. However, since the 1990s microscopy concepts have emerged providing diffraction-unlimited resolution by inhibiting the fluorescence of the dye such that features closer than the diffraction limit Δ*r* are forced to fluoresce sequentially.[1, 2] Depending on how this fluorescence inhibition is implemented, the techniques broadly fall into two groups. In the group encompassing stimulated emission depletion (STED) microscopy,[2] the coordinate where the fluorophores are allowed to fluorescence is predetermined by a pattern of light in which the intensity reaches zero at a controllable position in space; in STED microscopy this light pattern typically has a doughnut shape. The second group of techniques enables the emission of fluorophores stochastically in space, such that just a single fluorophore is able to emit within a region of diameter Δ*r=λ*/(2ΝΑ); the random emission coordinate is found by imaging the fluorescence with a camera, and then performing a centroid calculation.[3, 4] In both groups, images below the diffraction limit are obtained by consecutively allowing a representative number of dye molecules to fluoresce.[1]

While most of these techniques have been applied to biological systems including DNA, high quality nanoscopy of DNA molecules has remained elusive.[5–7] This situation is unfortunate because many of DNA's functions, such as gene expression, are known to be regulated by bending, looping, supercoiling, and other conformational changes at subdiffraction length scales.[8] Many conformational changes of DNA appear in the range of 100–1000 basepairs, approximately 35–350 nm, with the persistence length of DNA (typically around 50 nm) defining a fundamental length scale. Additionally, to study the conformational changes and variations present in DNA, the given structure has to be not only uniformly labeled but also uniformly recorded. In particular, it is essential to be able to distinguish integral single strands of DNA from a strand that has been broken up into pieces or from multiple overlaid strands.[9]

These requirements for far-field optical nanoscopy of DNA strands stained with standard intercalating dyes, such as YOYO-1 (YOYO), suggest that the deterministic nature of STED nanoscopy may have an inherent advantage over the stochastic approach termed stochastic ground-state depletion followed by individual molecular return (GSDIM, later also called dSTORM).[10–12] Whereas stochastic techniques rely quadratically on the number of photons to localize an emitter with increased resolution, in STED nanoscopy a few photons from the sample are sufficient to identify a molecule. Also, for the stochastic methods, the localization accuracy decreases for slightly defocused dyes with fixed dipole moment,[13] which could be relevant for YOYO molecules, the transition dipole moment of which is linked to the helical pitch of the DNA by intercalation.[14] Moreover, the depletion of the ground state underlying GSDIM entails pumping the dye to a more reactive state,[10–12] potentially harming or breaking the DNA strand (e.g. through electron transfer).[5–7] In contrast, STED is designed to disallow excited states, thus protecting the molecule from photoreactions.[15] Last but not least, to ensure that all but one of the fluorophores are transferred to a dark state within a diffraction limited volume in GSDIM, the dye concentration has to be matched to the lifetime of the dark-state. Fulfilling this condition is challenging because the dye can assume a wide range of dark states along the DNA strand, featuring a broad spectrum of lifetimes.[5–7] Not matching them, results in discontinuously imaged DNA strands and hence in unreliable information about DNA conformation. This problem is especially true for DNA bending and looping points, where nanoscale resolution is critical. For all these reasons, we decided to explore STED nanoscopy for imaging single DNA molecules.

STED nanoscopy was performed by overlaying a pulsed excitation beam with a doughnut-shaped STED beam thus prohibiting the fluorescence of all the dye molecules exposed to the excitation light, except those lying within the center of the doughnut. Scanning the interlocked beams across the sample makes the object details fluoresce sequentially. Images were taken using two different pulsed wavelengths (568 nm and 647 nm) for STED. The asymmetrical dimeric cyanine dye, YOYO, is often used for single-molecule DNA studies owing to its brightness and its fluorescence enhancement (ca. 500-fold) upon DNA binding. On the other hand, intercalating cyanines tend to promote photodamage of the DNA–dye complex, manifested by elevated bleaching and breaking (photonicking) of the DNA. Photonicking can be drastically reduced by removing oxygen in the buffer but the effect of oxygen on photobleaching remains unclear, although oxidation of DNA basepairs is believed to contribute to the observed bleaching.[16] We found that adding β-mercaptoethanol (BME) was effective in preventing both photonicking and bleaching. In the STED recordings, photostability was found to be highest for 20–50 photon counts per pixel (pixel size ca. 25 nm) at a pixel dwell time of 100 μs.

Using STED at 568 nm we obtain a five- to sixfold improvement in resolution over standard confocal microscopy (Figure 1) that in turn already provides a marked improvement in contrast and resolution over epifluorescence microscopy (Figure 2 c). In Figure 1, note the excellent correspondence of the variation in intensity along the DNA strands between the STED and confocal images. To explore the range of STED wavelengths that can be applied in our system we also used STED at 647 nm where the YOYO emission is a mere 3 % of its maximum. The result is a three- to fourfold improvement in resolution over standard confocal microscopy (Figure 2), thus demonstrating the applicability of STED over a range of over 80 nm. Kinks occur along DNA and can be sequence specific or due to the binding of proteins or small molecules. Figure 2 shows how STED, but not confocal microscopy can readily be used to identify these subtle structures along the DNA.

**Figure 1:**
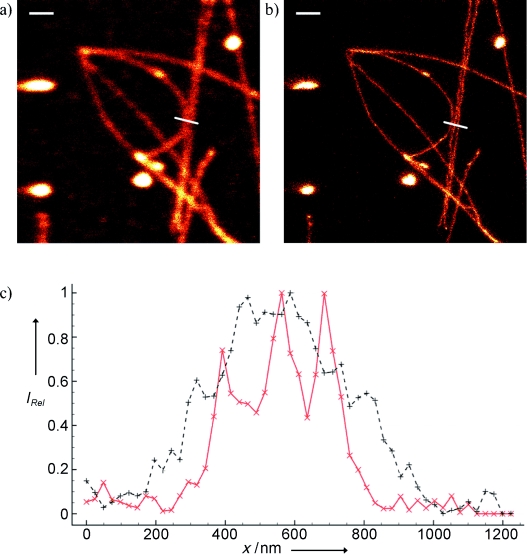
a) Confocal image of YOYO stained λ-DNA (basepair:dye 5:1). b) The corresponding STED image taken with λ_STED_=568 nm (raw data). The STED image was acquired before the confocal counterpart. Scale bars: 1 μm. c) Average of three line profiles from the STED (solid red line) and confocal (dotted black line) images. Line profiles extracted along the white lines in (a) and (b). The three distinct peaks belonging to different DNA molecules are only resolved by STED.

**Figure 2:**
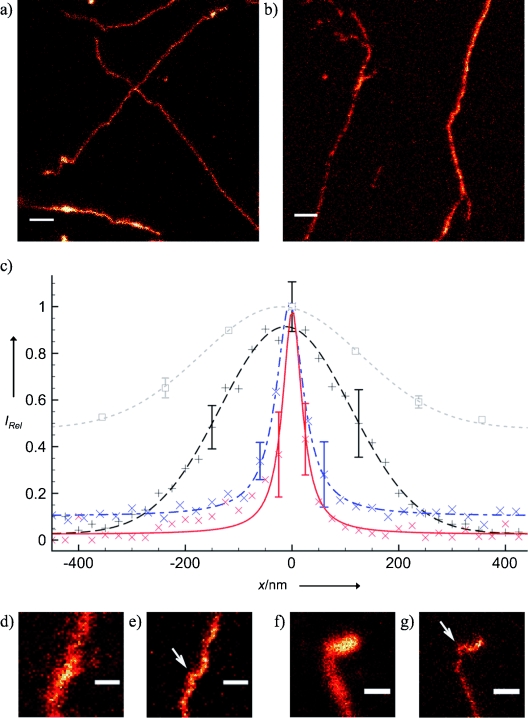
Typical raw STED images of YOYO stained λ-DNA (basepair:dye 5:1) using a) λ_STED_=568 nm and b) λ_STED_=647 nm. Scale bars in (a) and (b): 1 μm. c) Graph showing the average of 11 line profiles of a single DNA strand, with fits, for standard epifluorescence (dotted light gray line), confocal (dashed black line), and STED nanoscopy with *λ*_STED_=647 nm (dash-dotted blue line) and 568 nm (solid red line); the corresponding full width at half maximum (FWHM) were found to be (300±11) nm (Gaussian), (238±5) nm (Gaussian), (62±2) nm (Lorentzian), and (42±3) nm (Lorentzian), respectively. The error bars correspond to one standard deviation. d)–g) Examples of DNA segments with bends and kinks visible (indicated by white arrows) in a STED image using e) λ_STED_=647 nm and g) λ_STED_=568 nm but not resolvable in the corresponding confocal images (d) and (f). Scale bars in (d)–(g): 500 nm.

To investigate the photodamage inflicted by the STED beam on the DNA–dye complex (basepair:dye 5:1), a confocal image, an STED image (λ_STED_=568 nm), and then a confocal image were acquired one after another. While the second confocal image displayed a (50±9) % lower fluorescence level because of bleaching, photonicking was not observed, neither in the STED nor in the second confocal recording. Another series with three consecutive confocal images revealed a reduction of the fluorescence level by (34±16) %. Thus with STED the difference to photobleaching from standard confocal imaging is not significant. For details regarding imaging using a lower dye ratio (compatible with single-molecule investigations of DNA–enzyme interactions) see Supporting Information.

In conclusion, we have demonstrated STED nanoscopy for DNA imaging at a resolution of approximately 45 nm, which is comparable to the persistence length, the fundamental length scale of the polymer physics of DNA. The variation in fluorescence signal over the DNA molecule corresponds well with that obtained by confocal microscopy, demonstrating the viability of STED for imaging single DNA molecules and a future potential use for comparison of signal variations caused by sequence specific dye binding or partial melting. The demonstrated combination of resolution and uniformity of imaging along the DNA strand is critical for visualizing small conformational changes as well as for optical mapping of DNA.[9] Importantly, STED can be applied over a relatively large wavelength range (at least 80 nm), with longer wavelengths being generally less prone to inducing photodamage, while still providing a marked resolution improvement. By employing molecular transitions between the two most basic states of a fluorophore, that is, the ground and the first electronically excited state, we anticipate STED will become the preferred optical pathway to exploring DNA at the molecular level.

## Experimental Section

λ-bacteriophage DNA (Amersham Biosciences, UK) was stained with YOYO-1 (Invitrogen, USA) to obtain the basepair:dye ratios of 5:1 and 20:1. Prior to experiments the stained DNA was diluted to 1 μg mL^−1^ using degassed 0.5×tris-borate-EDTA (TBE) buffer containing 5 v/v % β-mercaptoethanol (Sigma Aldrich Corp., USA) and stretched on poly-l-lysine coated glass slides. For more details see Supporting Information.

Excitation of DNA was performed using a pulsed laser diode (Picoquant, Germany) emitting at λ_exc_=470 nm, with a peak irradiation of 15–65 kW cm^−2^ in the focal plane (time-average power of 1–4 μW), synchronized with a STED-laser at λ_STED_=568 nm and 647 nm by a fast photodiode (OCF-401, Becker & Hickl GmbH, Germany). STED was performed using an actively mode-locked (APE, Germany) krypton laser (Coherent Inc., USA) creating pulse widths of 1.5 ns (568 nm) and 300 ps (647 nm) at a repetition rate of 71.25 MHz and peak irradiations of 20–30 MW cm^−2^ (568 nm) and 210–360 MW cm^−2^ (647 nm) in the focal plane (time-average power of 45–70 mW (568 nm) and 130–220 mW (647 nm)). A vortex phase plate (RPC Photonics, USA) was used to generate a doughnut-shaped focal spot for STED. The excitation and STED beams were combined using acousto-optical tunable filters (Crystal Technologies, USA) and coupled into a microscope stand (DMI 4000B, Leica Microsystems GmbH, Germany) equipped with a 63× (NA 1.30, Leica) oil immersion objective and a three-axis piezo stage-scanner (PI, Germany). The emitted fluorescence passed through a band-pass filter (HQ510/40M, Chroma, USA) and was detected confocally with an avalanche photo diode (SPCM-AQR-13-FC, PerkinElmer Inc., USA) using a data acquisition software (Imspector, MPI Göttingen, Germany). Pixel sizes of 25 nm for λ_STED_=568 nm and 40 nm for λ_STED_=647 nm were chosen at a pixel dwell time of 100 μs. The corresponding confocal images were recorded using the same parameters.

## References

[1] Hell SW (2007). Science.

[2] Hell SW, Wichmann J (1994). Opt. Lett..

[3] Rust MJ, Bates M, Zhuang XW (2006). Nat. Methods.

[4] Betzig E, Patterson GH, Sougrat R, Lindwasser OW, Olenych S, Bonifacino JS, Davidson MW, Lippincott-Schwartz J, Hess HF (2006). Science.

[5] Flors C, Ravarani NJ, Dryden DTF (2009). ChemPhysChem.

[6] Flors C (2011). Biopolymers.

[7] Flors C (2010). Photochem. Photobiol. Sci..

[8] Vilar JMG, Saiz L (2005). Curr. Opin. Genet. Dev..

[9] Dimalanta ET, Lim A, Runnheim R, Lamers C, Churas C, Forrest DK, de Pablo JJ, Graham MD, Coppersmith SN, Goldstein S, Schwartz DC (2004). Anal. Chem..

[10] Bock H, Geisler C, Wurm CA, Von Middendorff C, Jakobs S, Schonle A, Egner A, Hell SW, Eggeling C (2007). Appl. Phys. B.

[11] Fölling J, Bossi M, Bock H, Medda R, Wurm CA, Hein B, Jakobs S, Eggeling C, Hell SW (2008). Nat. Methods.

[12] Heilemann M, van de Linde S, Schuttpelz M, Kasper R, Seefeldt B, Mukherjee A, Tinnefeld P, Sauer M (2008). Angew. Chem..

[13] Engelhardt J, Keller J, Hoyer P, Reuss M, Staudt T, Hell SW (2011). Nano Lett..

[14] Persson F, Westerlund F, Tegenfeldt JO, Kristensen A (2009). Small.

[15] Hotta J, Fron E, Dedecker P, Janssen DPF, Li C, Müllen K, Harke B, Bückers J, Hell SW, Hofkens J (2010). J. Am. Chem. Soc..

[16] Kanony C, Akerman B, Tuite E (2001). J. Am. Chem. Soc..

