# Supplementary material for: Fluorescence Nanoscopy of Single DNA Molecules by Using Stimulated Emission Depletion (STED)
Source: Angew Chem Int Ed Engl. 2011 May 6;50(24):5581–3. doi: 10.1002/anie.201100371 (PMC3229986; doi:10.1002/anie.201100371)
Supplement: Supplementary file 1 [file anie0050-5581-SD1.pdf]

Angewandte Chemie
Eine Zeitschrift der Gesellschaft Deutscher Chemiker

Supporting Information
© Wiley-VCH 2011
69451 Weinheim, Germany

Fluorescence Nanoscopy of Single DNA molecules by using Stimulated Emission Depletion (STED)**

*F. Persson, P. Bingen, T. Staudt, J. Engelhardt, J. O. Tegenfeldt, and Stefan W. Hell**

anie_201100371_sm_miscellaneous_information.pdf

Supporting Information

Experimental Details

Sample preparation: Coverslides were cleaned with methanol and blowdried. A 50 µl droplet of poly-L-lysine solution (Sigma Aldrich Corp., St. Louis, USA) was deposited on the coverslides. After 5 min the slides were rinsed with Milli-Q water and blowdried. The DNA was prepared by mixing λ-bacteriophage DNA (Amersham Biosciences, Buckinghamshire, UK) at a concentration of 10 µg/ml (diluted using 0.5x TBE buffer) with YOYO-1 (Invitrogen, Carlsbad, USA) in order to obtain the desired basepair:dye staining ratio of 5:1 and 20:1. Prior to the experiments this solution of labelled DNA was diluted 10-fold with an imaging buffer consisting of 0.5x TBE buffer, degassed under ultrasonic agitation for 30 min, and 5 v/v% BME. This solution was then deposited in 5 µl droplets on the slides and stretched using shear-combing or flow stretching. Imaging buffer was added to prevent the sample from drying.

Imaging using a lower dye ratio (20:1)

Photodamage, including photobleaching of the dye/DNA complex and photonicking of the DNA, depends on the basepair:dye ratio. It has also been shown that YOYO at high concentration on DNA can inhibit enzymatic function. Due to this, STED and confocal images were also recorded on DNA labelled with a basepair:dye ratio of 20:1, demonstrated not to inhibit the activity of λ-exonuclease.[1]

Multiple consecutive STED (λ_{STED}=647 nm) and confocal images could routinely be acquired without any observed photonicking appearing during imaging (see Fig. S1 – S5). In all images the resolution is consistent to that achieved with 5:1 labelling and small kinks and individual strands can readily be discerned (see Fig. S1).

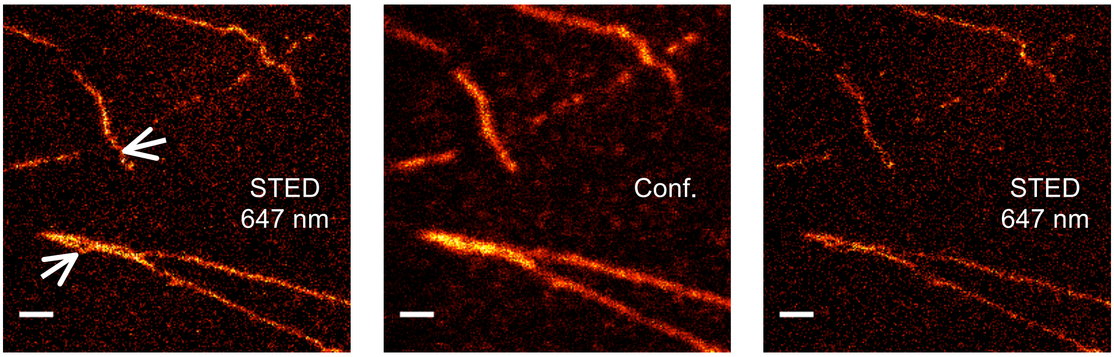

Figure S1: A series of 3 sequential images (1 confocal and 2 STED) of stretched DNA labeled with a basepair:dye ratio of 20:1, with the leftmost image acquired first. Note the fine details in the STED image that is not resolvable in the corresponding confocal image (white arrows). Scale bars correspond to 1 µm.

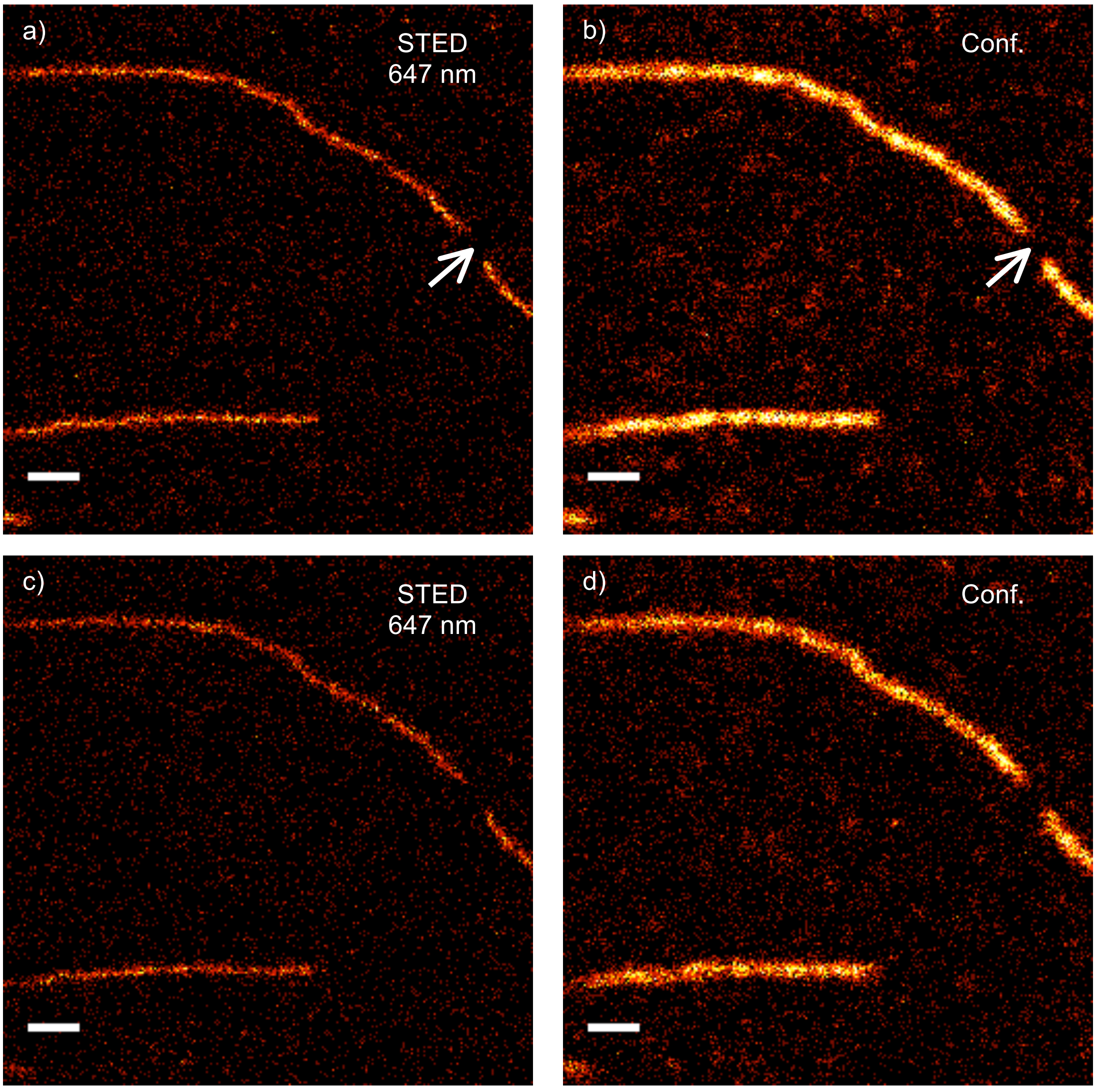

Figure S2: A series of 4 sequential images (2 confocal and 2 STED) of stretched DNA labeled with a basepair:dye ratio of 20:1. The images are acquired in alphabetical order. Note that strand breaks, typically occurring in photonicking events, can be detected. Scale bars correspond to 1 µm.

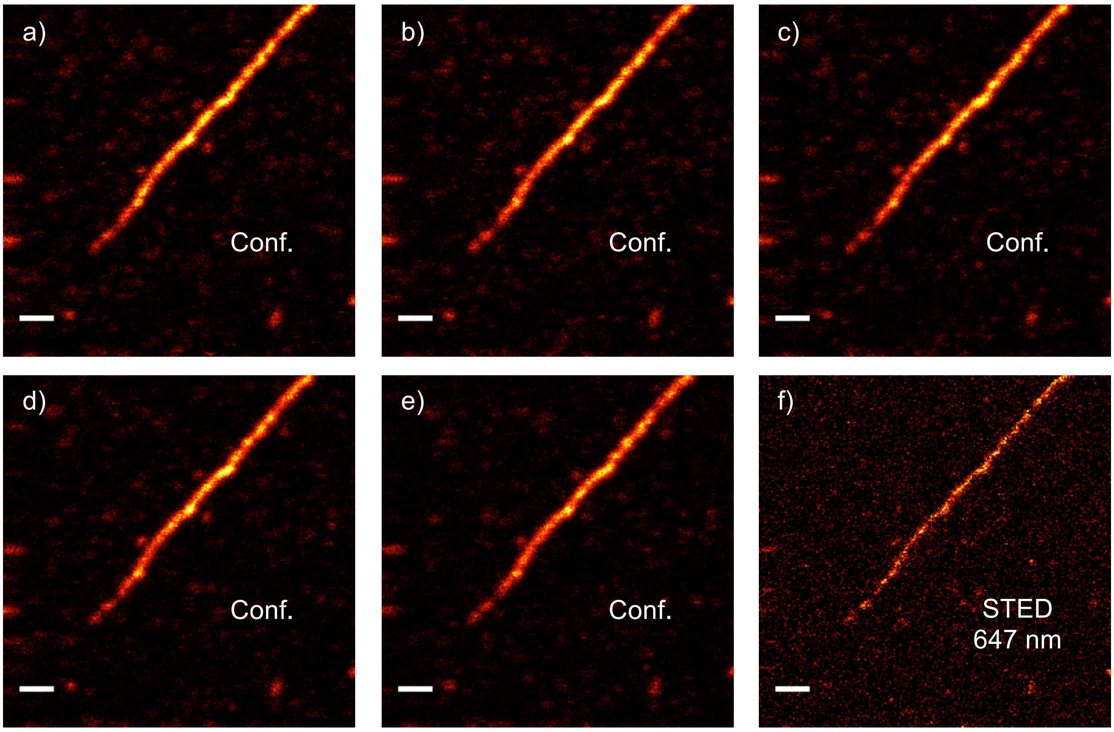

Figure S3: A series of 6 sequential images (5 confocal and 1 STED) of stretched DNA labeled with a basepair:dye ratio of 20:1. The images are acquired in alphabetical order. Scale bars correspond to 1 µm.

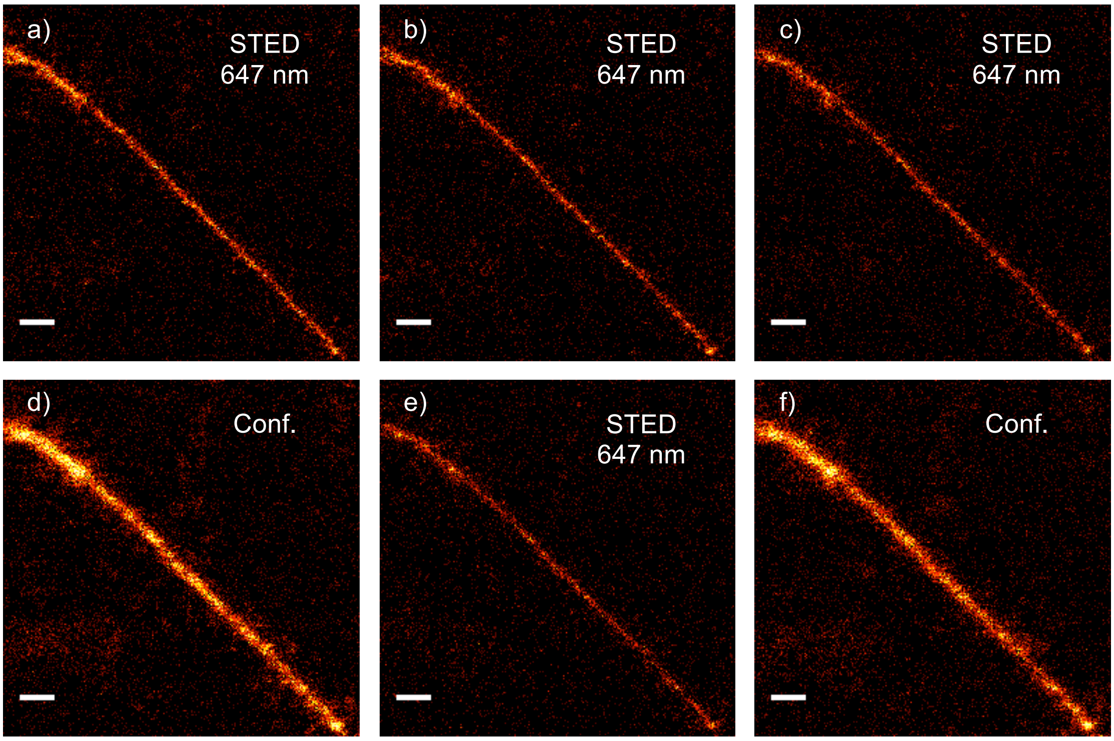

Figure S4: A series of 6 sequential images (2 confocal and 4 STED) of stretched DNA labeled with a basepair:dye ratio of 20:1. The images are acquired in alphabetical order. Note the absence of visible photonicking events. Scale bars correspond to 1 µm.

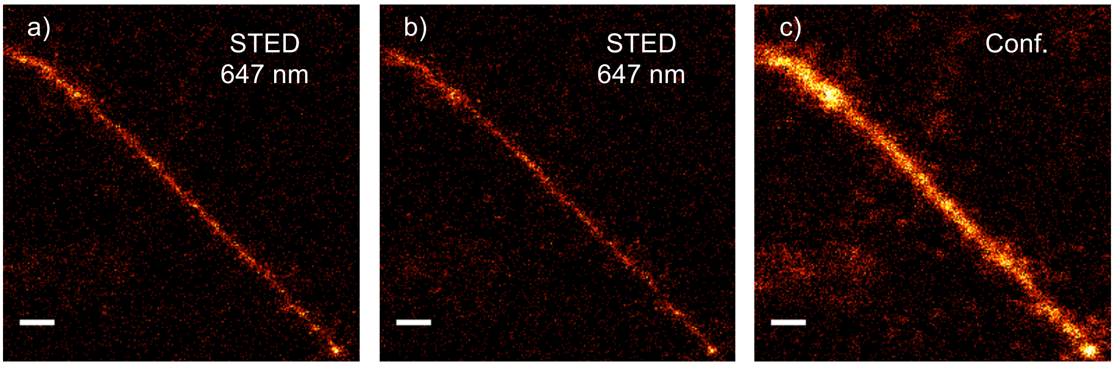

Figure S5: A series of 3 sequential images (1 confocal and 2 STED) taken directly after those in Figure S4 (corresponding to images 7-9 in the same image series). The images are acquired using an increased STED and excitation power compared to those in Figure S4. The images are acquired in alphabetical order. Note the absence of visible photonicking events. Scale bars correspond to 1 μm.

References:

[1] S. Matsuura, J. Komatsu, K. Hirano, H. Yasuda, K. Takashima, S. Katsura, A. Mizuno, Nucleic Acids Res. 2001, 29, e79.
